# Preexposure Prophylaxis Modality Preferences Among Men Who Have Sex With Men and Use Social Media in the United States

**DOI:** 10.2196/jmir.5713

**Published:** 2016-05-19

**Authors:** Eric William Hall, Walid Heneine, Travis Sanchez, Robert Craig Sineath, Patrick Sullivan

**Affiliations:** ^1^ Department of Epidemiology Rollins School of Public Health Emory University Atlanta, GA United States; ^2^ National Center for HIV/AIDS, Viral Hepatitis, STD, and TB Prevention Centers for Disease Control and Prevention Atlanta, GA United States

**Keywords:** preexposure prophylaxis, PrEP, men who have sex with men, MSM, HIV prevention, Facebook, survey

## Abstract

**Background:**

Preexposure prophylaxis (PrEP) is available as a daily pill for preventing infection with the human immunodeficiency virus (HIV). Innovative methods of administering PrEP systemically or topically are being discussed and developed.

**Objective:**

The objective of our study was to assess attitudes toward different experimental modalities of PrEP administration.

**Methods:**

From April to July 2015, we recruited 1106 HIV-negative men who have sex with men through online social media advertisements and surveyed them about their likelihood of using different PrEP modalities. Participants responded to 5-point Likert-scale items indicating how likely they were to use each of the following PrEP modalities: a daily oral pill, on-demand pills, periodic injection, penile gel (either before or after intercourse), rectal gel (before/after), and rectal suppository (before/after). We used Wilcoxon signed rank tests to determine whether the stated likelihood of using any modality differed from daily oral PrEP. Related items were combined to assess differences in likelihood of use based on tissue or time of administration. Participants also ranked their interest in using each modality, and we used the modified Borda count method to determine consensual rankings.

**Results:**

Most participants indicated they would be somewhat likely or very likely to use PrEP as an on-demand pill (685/1105, 61.99%), daily oral pill (528/1036, 50.97%), injection (575/1091, 52.70%), or penile gel (438/755, 58.01% before intercourse; 408/751, 54.33% after). The stated likelihoods of using on-demand pills (median score 4) and of using a penile gel before intercourse (median 4) were both higher than that of using a daily oral pill (median 4, *P*<.001 and *P*=.001, respectively). Compared with a daily oral pill, participants reported a significantly lower likelihood of using any of the 4 rectal modalities (Wilcoxon signed rank test, all *P*<.001). On 10-point Likert scales created by combining application methods, the reported likelihood of using a penile gel (median 7) was higher than that of using a rectal gel (median 6, *P*<.001), which was higher than the likelihood of using a rectal suppository (median 6, *P*<.001). The modified Borda count ranked on-demand pills as the most preferred modality. There was no difference in likelihood of use of PrEP (gel or suppository) before or after intercourse.

**Conclusions:**

Participants typically prefer systemic PrEP and are less likely to use a modality that is administered rectally. Although most of these modalities are seen as favorable or neutral, attitudes may change as information about efficacy and application becomes available. Further data on modality preference across risk groups will better inform PrEP development.

## Introduction

Although improvements in treatment have extended the life expectancy of people infected with the human immunodeficiency virus (HIV), there are still a troubling number of new HIV infections each year. In particular, HIV incidence rates among men who have sex with men (MSM) are increasing in North America and several other regions of the world [1,2]. In 2014, 70% of all new infections in the United States occurred among MSM [3]. To reduce the number of new infections, prevention strategies targeted toward specific risk groups are needed.

A recent and exciting strategy, HIV preexposure prophylaxis (PrEP), involves using antiretroviral medication to reduce the risk of HIV infection among HIV-negative individuals. In 2010, this concept was first demonstrated in humans with publication of results from the iPrEx study, a randomized controlled trial that tested the efficacy of tenofovir disoproxil fumarate (TDF) in combination with emtricitabine (FTC) among MSM [4]. The iPrEx trial demonstrated a 44% (95% CI 15%-63%) reduction in HIV incidence among men who were taking TDF/FTC as a daily oral pill compared with a placebo group. Since then, other randomized controlled trials have indicated that a daily PrEP pill can reduce risk of HIV transmission in HIV-discordant couples [5], sexually active heterosexual men and women [6], and intravenous drug users [7]. However, 2 other trials showed no protective effect in heterosexual women in high-risk areas of Africa, but both of these studies had problems with adherence [8,9].

Results from these PrEP efficacy studies show varying degrees of HIV risk reduction that ranges from 0% to 75%. However, this wide range is most commonly attributed to varying levels of adherence, because PrEP efficacy is much higher among participants who demonstrated consistent use [10-14]. For example, when blood samples were analyzed in the iPrEx study, HIV incidence reduction was 92% among participants who had the drug detectable in their blood and 99% among participants who had drug levels corresponding to daily use (both compared with the placebo group) [15,16].

In 2014, the US Centers for Disease Control and Prevention and the World Health Organization released guidelines that recommend the use of daily oral PrEP in populations with an elevated risk of HIV infection [17,18]. However, barriers such as cost, the burden of taking a daily pill, and concerns about potential health effects (both long-term and short-term effects) have led to PrEP being underused by eligible people [13,19-21].

Although a daily oral TDF/FTC pill is the only approved and recommended form of PrEP, there is growing interest in developing new methods for administering antiretroviral drugs as prevention. Topical applications of PrEP have been studied in 2 clinical trials of high-risk women who used a TDF-based vaginal gel either before and after sex [22] or on a daily basis [8]. Although the results from these trials present conflicting conclusions, further analysis indicates that the efficacy of vaginal gel depends on the concentration of tenofovir in the cervicovaginal fluid, which is also likely an indication of adherence [23]. A recent study among MSM (iPERGAY) investigated the efficacy of intermittent TDF/FTC pills taken just before and after sexual encounters, but the study was stopped early because initial analysis found comparable protection against HIV infection between these on-demand PrEP regimens and daily PrEP [24].

Researchers are investigating the delivery of PrEP as oral pills that are used intermittently (ie, less than a daily basis), topical gels, vaginal rings, and long-lasting injections [21,25,26]. Because high levels of PrEP efficacy are dependent on adherence, there is obvious interest in developing administration modalities that target groups are willing to use. To help guide these research efforts, we sought to assess attitudes among MSM toward a variety of potential modalities of PrEP administration.

## Methods

### Recruitment

We collected data through a study that primarily explored alternative methods for delivering consent information and maximizing retention in online surveys [27] (funded by NICHD Research Grant 1R21-HD074502-01A1). Participants were recruited through targeted advertisements on a social media website (Facebook) from April 2015 to July 2015. Recruitment advertisements appeared to users in the United States who indicated on their Facebook profile that they are male, over 18 years of age, and interested in men. People who clicked on the advertisements were directed to an online consent module and a short screener to determine eligibility. To be eligible for the survey, users had to be male, between 18 and 34 years old, and not report having sex only with women in the past year. Men who reported never having oral or anal sex with a man were removed from the analysis dataset.

Eligible men were given an online survey that collected demographic information such as age, education, race or ethnicity, zip code, and self-identified sexual orientation. The survey also collected information about sexual history and current sexual practices, history of HIV testing, and relationship status. All study materials and procedures were approved by the Emory University Institutional Review Board.

Participants who reported a negative or unknown HIV status were asked about their knowledge of PrEP and history of use. Participants who had not previously used PrEP answered 5-point Likert-scale items that asked how likely they were to use different PrEP modalities to reduce the risk of getting HIV. They were asked about 9 Likert-scale items, 1 for each of the modalities listed in Table 1. Participants were only asked about modalities that involved penile application if they reported having insertive anal sex in the past year. Likewise, participants were only asked about the rectal modalities if they reported having receptive anal sex in the past year. We collected responses to each Likert-scale item in the following format: 1=very unlikely, 2=somewhat unlikely, 3=neither likely or unlikely, 4=somewhat likely, 5=very likely.

**Table 1:** Preexposure prophylaxis modalities presented^a^ to online survey respondents aged 18–34 years, by type of anal sex with male partner(s) in the past year, United States, April–July 2015.

Modalities	Insertive only	Receptive only	No anal sex
Daily oral pill	X^b^	X	X
On-demand pills^c^	X	X	X
Injection every 1–3 months	X	X	X
Penis gel before insertive intercourse	X		
Penis gel after insertive intercourse	X		
Rectal gel before receptive intercourse		X	
Rectal gel after receptive intercourse		X	
Rectal suppository 30 minutes before receptive intercourse		X	
Rectal suppository 3 hours after receptive intercourse		X	

^a^The survey included individual Likert-scale items asking the likelihood of using each modality.

^b^Participants who indicated they had both insertive and receptive anal sex in the past year were presented all modalities. Modalities presented to participants who said they only had insertive anal sex, receptive anal sex, or no anal sex in the past year are indicated by an “X”. Depending on their response, participants were then presented with a complete list (for each sexual behavior group) and asked to rank the modalities from most likely to least likely to use.

^c^Includes 2 pills within 24 hours before sex and 2 separate 1-pill doses within 2 days after sex.

Participants were also asked to rank their interest in using each of the different potential methods of PrEP administration. The number of modalities that each participant could rank depended on the type of sex he indicated having in the past 12 months. If a respondent said he had both insertive and receptive anal sex in the past year, he could rank all 9 potential modalities. However, if a respondent indicated he only had receptive anal sex in the past 12 months, he was prompted to rank only 7 potential modalities (4 that are applied rectally, 2 applied orally, and an injection).

### Statistical Analyses

We did all analyses using SAS v9.4 (SAS Institute Inc). Participants who did not respond to any of the PrEP modality Likert-scale items (n=318) were removed from the analysis dataset. We summarized the likelihood of using each PrEP modality by finding the mean, median, and mode of the 5-point Likert-scale item response. Since these items are ordinal and not interval data, we used nonparametric tests for statistical inferences. We used Wilcoxon signed rank tests to determine whether the reported likelihood of using each of the 8 experimental PrEP modalities differed from the likelihood of using PrEP as a daily oral pill. Each test was considered statistically significant at alpha=.05.

Individual Likert-scale items that asked about topical application sites were summed to create separate Likert scales based on application method and time of application. This resulted in three 10-point Likert scales based on method (penile gel, rectal gel, and rectal suppository) and two 15-point Likert scales based on time of application (before intercourse, after intercourse) [28].

To assess demographic associations with likelihood of using each modality, we dichotomized individual Likert-scale item responses so that “somewhat likely” or “very likely” indicated likelihood of use (versus “somewhat unlikely” or “very unlikely”). Responses of “neither likely or unlikely” were set to missing. We used logistic regression to determine unadjusted odds ratios (ORs) and corresponding confidence intervals for demographic variables of interest.

We used the modified Borda count method to determine the order of preference for the different PrEP modalities [29]. Each modality was assigned a number of points that corresponded to the position in which it was ranked by the participant. The number of points given to a participant’s first choice was equal to the number of modalities he actually ranked. We then summed points for each modality to create a collective ranking. Since the number of options presented to an individual depended on the type of sex he reported in the past 12 months (eg, a participant who only reported insertive anal sex was not presented with modalities that are administered rectally), we stratified cumulative rankings by reported sexual practices.

## Results

There were 3990 participants who started the online survey and answered eligibility questions. Of 1921 men who met the eligibility requirements for the survey, 4 reported having tested positive for HIV and 493 reported never having oral or anal sex with a man. We further limited the final analysis dataset to the 1106 participants who answered at least one of the questions related to PrEP.

Table 2 summarized the demographic characteristics of the 1106 participants included in the analysis. Almost half of the participants (542/1106, 49.01%) were between 18 and 24 years old and the mean age was 25.2 years. The highest proportion (800/1106, 72.33%) of participants were white, and most had received some level of education past secondary school (1025/1106, 92.68%). The highest proportion of respondents lived in the south (392/1106, 35.44%) but there were at least 200 respondents from all 4 US census regions. The majority of participants identified as homosexual (965/1106, 87.25%) and reported having had anal sex with a male partner in the past month (964/1106 87.16%). In the previous 12 months, 599 (54.16%) participants reported having both insertive and receptive anal sex with a male partner, 202 (18.26%) reported having only insertive sex, and 163 (14.74%) reported having only receptive sex. There were 138 (12.48%) participants who did not have anal sex in the previous 12 months. The majority of participants (824, 74.50%) had previously heard of people using PrEP to reduce the risk of getting HIV.

**Table 2:** Demographic and sexual behavior characteristics of 1106 men who have sex with men, aged 18–34 years, participating in an online survey about preexposure prophylaxis (PrEP) for HIV infection, United States, April 2015–July 2015.

Characteristics		n	(%)
**Age (years)**^a^			
	18–24	542	(49.01)
	25–29	334	(30.20)
	30–34	230	(20.80)
**Race/ethnicity**			
	White, non-Hispanic	800	(72.33)
	Black, non-Hispanic	51	(4.61)
	Hispanic	181	(16.37)
	Other	74	(6.69)
**US census region**			
	Midwest	217	(19.62)
	Northeast	251	(22.69)
	South	392	(35.44)
	West	227	(20.52)
	Unknown	19	(1.72)
**Highest level of education**			
	High school or less	80	(7.23)
	Some college, associate degree, or technical degree	306	(27.67)
	Bachelor degree	384	(34.72)
	Any graduate or professional school	335	(30.29)
	Unknown	1	(0.09)
**Sexual identity**			
	Homosexual	965	(87.25)
	Bisexual	106	(9.58)
	Other^b^	35	(3.16)
**Type of anal sex with male partner(s) in past 12 months**			
	Insertive only	202	(18.26)
	Receptive only	163	(14.74)
	Both insertive and receptive	599	(54.16)
	No anal sex	138	(12.48)
**Previously heard of PrEP**		824	(74.50)

^a^Mean 25.2 years, median 25 years, range 18–34 years.

^b^There were 5 participants who indicated heterosexual, 15 indicated unsure, 14 indicated other, and 1 who did not indicate sexual identity.

**Figure 1:**
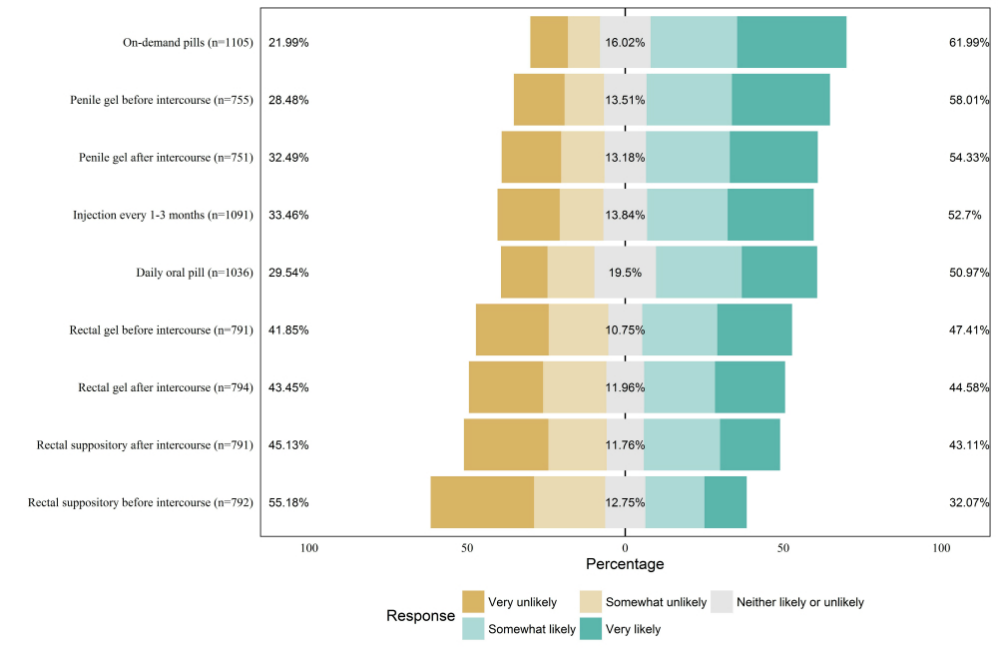
Stated likelihood of using different preexposure prophylaxis modalities among 1106 men who have sex with men, aged 18-34 years, participating in an online survey, United States, April–July 2015.

Figure 1 displays the stated likelihood of using each PrEP modality to reduce the risk of contracting HIV. Overall, over half of participants stated they would be somewhat likely or very likely to use on-demand pills (685/1105, 61.99%), penile gel (438/755, 58.01% before intercourse and 408/751, 54.33% after intercourse), a periodic injection (575/1091, 52.70%), or a daily oral pill (528/1036, 50.97%). The majority (437/792, 55.18%) of respondents indicated they would be very unlikely or somewhat unlikely to use a rectal suppository before intercourse.

When the responses were analyzed on a 5-point scale, the mean responses ranged from 2.58 (rectal suppository before intercourse, median 2) to 3.63 (on-demand pills, median 4; Table 3). Compared to the currently available daily pills (median 4), respondents reported a higher likelihood of using on-demand pills (median 4, *P*<.001) or a penile gel before intercourse (median 4, *P*=.001). However, participants reported a significantly lower likelihood of using each of the rectal modalities (all *P*<.001) compared with a daily oral pill.

**Table 3:** Stated likelihood of using different preexposure prophylaxis modalities for HIV infection among 1106 men who have sex with men, aged 18–34 years, participating in an online survey, United States, April–July 2015.

Individual Likert-scale items for each modality^a^	n	Mean (SD)	Median (IQR^b^)	Mode	*P*-value^c^
Daily oral pill	1036	3.31 (1.4)	4 (2)	4	Reference
On-demand pills^d^	1105	3.63 (1.4)	4 (2)	5	<.001
Injection^e^	1091	3.27 (1.5)	4 (3)	5	.02
Penile gel before intercourse	755	3.45 (1.4)	4 (3)	5	.001
Penile gel after intercourse	751	3.31 (1.5)	4 (3)	5	.28
Rectal gel before intercourse	791	3.06 (1.5)	3 (2)	4	<.001
Rectal gel after intercourse	794	3.00 (1.5)	3 (2)	1	<.001
Rectal suppository before intercourse	792	2.58 (1.4)	2 (3)	1	<.001
Rectal suppository after intercourse	791	2.90 (1.5)	3 (3)	1	<.001

^a^The 5-point Likert-scale items where 1=very unlikely, 2=somewhat unlikely, 3=neither likely or unlikely, 4=somewhat likely, 5=very likely.

^b^IQR: interquartile range.

^c^ *P*-values calculated using Wilcoxon signed rank tests with alpha=.05.

^d^Consisting of 2 pills 24 hours before sex and 2 separate 1-pill doses after.

^e^Every 1–3 months.

On 10-point Likert scales created by combining modalities by topical application methods, the reported likelihood of using a penile gel (median 7) was higher than that of using a rectal gel (median 6, *P*<.001; Table 4). However, the likelihood of using a rectal gel was higher than that of using a rectal suppository (median 6, *P*<.001). There was no statistically significant difference in the reported likelihood of using a topical PrEP modality (gel or suppository) before or after intercourse.

When individual Likert-scale item responses were dichotomized, stated likelihood of using daily oral pills differed by race/ethnicity, age category, and highest level of education (Multimedia Appendix 1). Compared with white participants, black participants had higher odds of reporting a favorable likelihood of using the following modalities: daily oral pills (OR 3.10, 95% CI 1.35–7.13), penis gel before intercourse (OR 6.59, 95% CI 1.54–28.24), penis gel after intercourse (OR 2.82, 95% CI 1.05–7.59), rectal gel before intercourse (OR 4.28, 95% CI 1.73–10.61), rectal gel after intercourse (OR 2.52, 95% CI 1.14–5.58) and a suppository before intercourse (OR 2.69, 95% CI 1.26–5.77).

**Figure 2:**
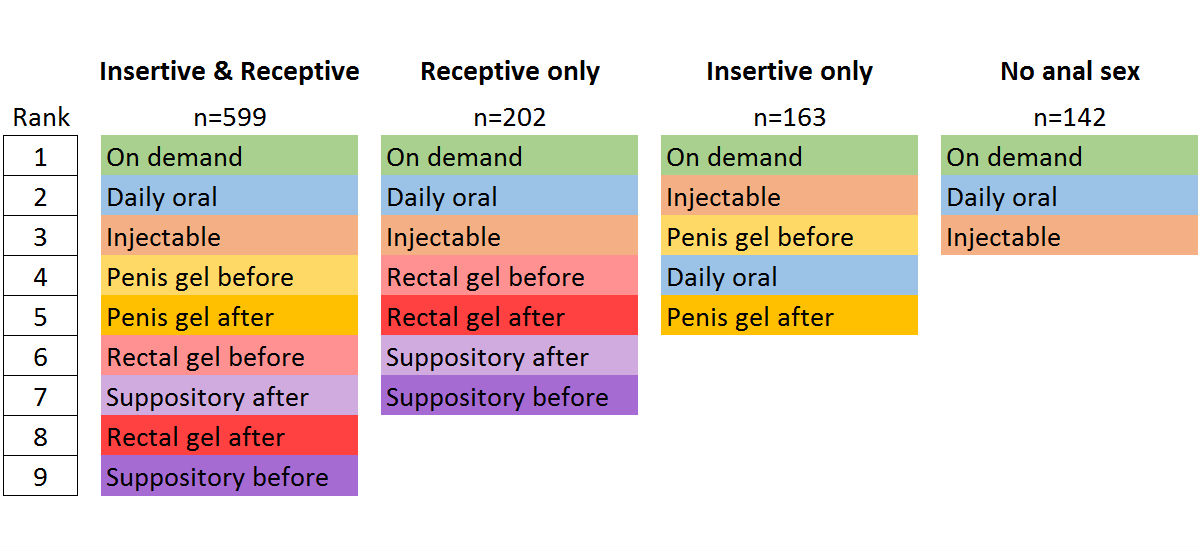
Modified Borda count ranking of different preexposure prophylaxis modalities, stratified by type of anal sex with a male partner in the past year, among 1106 men who have sex with men, aged 18–34 years, participating in an online survey, United States, April-July 2015. Respondents were presented with a different number of modalities to rank, depending on the type of anal sex they reported in the past 12 months.

Figure 2 shows the modified Borda count rankings, stratified by type of anal sex in the past 12 months. On-demand pills were the top-ranked modality for each sexual behavior group. In general, modalities administered orally were ranked highest and modalities administered rectally were ranked lowest.

**Table 4:** Stated likelihood of using different preexposure prophylaxis topical modalities, by method and time of application, among 1106 men who have sex with men, aged 18–34 years, participating in an online survey, United States, April–July 2015.

Combined Likert scales^a^		n	Mean (SD)	Median (IQR^b^)	Mode	*P*-value^c^
**Application method**^d^						
	Penile gel, anytime	750	6.76 (2.7)	7 (4)	10	<.001
	Rectal gel, anytime	790	6.06 (2.9)	6 (4)	2	Reference
	Rectal suppository, anytime	790	5.48 (2.8)	6 (6)	2	<.001
**Time of application**^e^						
	Before intercourse	585	8.87 (3.8)	9 (6)	3	Reference
	After intercourse	584	9.02 (4.0)	9 (6)	3	.14

^a^Created from original 5-point Likert-scale items where 1=very unlikely, 2=somewhat unlikely, 3=neither likely or unlikely, 4=somewhat likely, 5=very likely.

^b^IQR: interquartile range.

^c^ *P*-values calculated using Wilcoxon signed rank tests with alpha=.05.

^d^Based on a 10-point scale created by adding the two 5-point Likert-scale items for each application method.

^e^Based on a 15-point scale created by adding the three 5-point Likert-scale items for each time of application.

## Discussion

Our findings indicate that 51% of MSM would be likely to take PrEP as a daily oral pill, which is consistent with the range seen in previous studies in which between 46.1% and 78.5% of MSM said they would be willing to use daily oral PrEP [19,30-33]. The varying range of acceptability is likely a result of some studies stipulating specific scenarios in which PrEP is either offered free of charge, does not cause side effects, or is 80% effective against preventing HIV infection. Some of this variation in acceptability could be related to when the survey was conducted in relation to the public release of the iPrEx results [4]. While our analysis did not investigate reasons against taking PrEP, previous research has indicated that concerns about health (both long-term consequences and immediate side effects), unknown efficacy, possibility of developing drug resistance, cost, and risk perception can all be barriers to use [13,19,20].

These results provide comparative insights on how likely MSM in our sample were to use a variety of hypothetical PrEP modalities. Overall, attitudes toward using PrEP to reduce the risk of contracting HIV were generally neutral or favorable, with a reported likelihood of use ranging from 32% (rectal suppository before intercourse) to 62% (on-demand pills). However, there are some noticeable differences in the likelihoods of using each modality.

The preference for intermittent oral PrEP is evident across this analysis. The highest proportion of participants indicated they would use on-demand pills, which was also the top consensus rank for each of the 4 modified Borda count groups. This preference is particularly of interest when viewed with the growing evidence base demonstrating the efficacy of intermittent (ie, less than daily) oral PrEP [24]. Community surveys have shown that the majority of condomless anal intercourse events appear to be anticipated in advance or infrequent enough to make event-driven or time-driven PrEP regimens feasible [34-36]. Furthermore, Parsons et al recently found that MSM overestimate the likelihood of having sex and are much better at predicting when they would not have sex [37]. This has implications on counseling related to intermittent PrEP use. Parsons et al concluded that counseling messages should encourage individuals to skip a daily dose only when they are sure there is no chance they will have sex the following day. Several forms of slow-acting injectable PrEP are being studied [26], and our study indicates they may have similar acceptability to daily oral pills. For most of the modalities (daily oral pills, both penis gels, both rectal gels, and suppository before intercourse), our study indicated that black participants were more likely than white participants to use them.

It is important to note that attitudes toward topical PrEP modalities differ by administration site. Although gels applied to the penis were generally viewed as acceptable, the 4 rectal modalities were the only Likert-scale items in which more than half of participants reported that they were unlikely to use them. While the combined Likert scales indicated that rectal gels are seen more favorably than rectal suppositories, both were less likely to be used than penile gels. Most research conducted on PrEP in a gel form has focused on vaginal gels for women in Africa [8,22], but there is an ongoing phase 2 trial on rectal gel microbicides in MSM (MTN-017) [38]. Phase 1 research indicated that 75% to 100% of recipients found the experimental gels to be acceptable [39].

### Limitations

Several limitations need to be considered when interpreting these results. First, because Likert-scale items are not interval in nature, only the direction of preference can be determined. The magnitude of preference cannot be adequately determined or compared (ie, we cannot say “how much more likely” somebody is to use a single modality over another). Second, we asked participants to state their likelihood of using different modalities without any specifications of cost, efficacy, or possible side effects. As this information (specific to each modality) becomes available, we would expect the reported likelihood of use to change. Furthermore, we asked participants about different modalities of administration based on the type of sex they reported having in the past year. As a result, the attitudes toward any of the modalities administered rectally represent only those of the participants who recently received anal sex (and vice versa for penile application and insertive anal sex). There is potential for selection bias in the analysis dataset. We excluded 318 respondents from the analysis because they did not respond to any of the PrEP modality Likert-scale items. Those excluded respondents were more likely to be African American (8.2% vs 4.6%, *P*=.009) and more likely to report not having anal sex with a male in the past year (27.8% vs 12.5%, *P*≤.001), compared with our sample of 1106 participants. Third, our recruitment methods targeted social media users and our sample may not be representative of the general community of MSM.

Since there is not a reference population that can be used as a comparison to assess representativeness, there have not been any other studies that characterized sampling biases using online convenience samples. However, this approach is one of the most common in the field, is consistent with the body of published literature, and allows researchers to quickly collect behavior information on a large number of MSM [40]. Our advertisements targeted young-adult MSM in the United States, which limits the ability to generalize these results to other age groups, regions, or populations affected by the HIV epidemic. Likelihood and acceptability of use is likely to vary depending on perceived risk and cultural acceptability.

### Conclusion

Previous research has shown that PrEP has the potential to reduce risk of HIV transmission, but adherence is essential to ensure efficacy. In order to overcome the many barriers to PrEP uptake, we need to develop modalities that are feasible and likely to be used. Among this specific population of MSM in the United States, intermittent systemic approaches seem to be preferred. For topical methods, those that involve application on the penis are preferred over rectal application, and gels are preferred over suppositories. However, further analysis is needed to determine why people would not use particular modalities. Other studies similar to this one need to be carried out for other groups that will be targeted for the newer PrEP modalities. While there may not be a single PrEP modality that is used by everybody, our study and future ones like it can help determine which technology is most likely to be adopted by specific communities.

## Appendix

Multimedia Appendix 1 Odds ratios for demographic characteristics and stated likelihood of using different preexposure prophylaxis modalities among men who have sex with men participating in an online survey, United States, April-July 2015.

## Abbreviations

FTC: emtricitabine
HIV: human immunodeficiency virus
MSM: men who have sex with men
OR: odds ratio
PrEP: preexposure prophylaxis
TDF: tenofovir disoproxil fumarate
