# Supplementary material for: Preexposure Prophylaxis Modality Preferences Among Men Who Have Sex With Men and Use Social Media in the United States
Source: J Med Internet Res. 2016 May 19;18(5):e111. doi: 10.2196/jmir.5713 (PMC4909393; doi:10.2196/jmir.5713)
Supplement: Multimedia Appendix 1 [file jmir_v18i5e111_app1.pdf]

Multimedia Appendix 1. Odds ratios for demographic characteristics and stated likelihood of using different pre-exposure prophylaxis modalities among MSM participating in an online survey, United States, April-July 2015

**Characteristic**	**Odds Ratio[a] (95% CI)**	**Type 3 *P* value**	**Odds Ratio[a] (95% CI)**	**Type 3 *P* value**	**Odds Ratio[a] (95% CI)**	**Type 3 *P* value**
**Systemic Modalities**						
	Daily oral pills		Injection[b]		On-demand pills[c]	
**Race/Ethnicity**		0.011		0.458		0.372
White, not Hispanic	Reference		Reference		Reference	
Black, not Hispanic	3.10 (1.35-7.13)		1.05 (0.55-2.01)		1.45 (0.68-3.09)	
Hispanic	1.36 (0.91-2.03)		1.32 (0.92-1.91)		1.32 (0.87-1.99)	
Other	1.76 (0.95-3.25)		1.23 (0.71-2.11)		1.37 (0.74-2.54)	
**Age (years)**		0.015		0.708		0.158
18-24	1.52 (1.05-2.20)		1.05 (0.75-1.48)		1.04 (0.70-1.54)	
25-29	1.00 (0.68-1.49)		1.16 (0.80-1.68)		0.76 (0.50-1.15)	
30-34	Reference		Reference		Reference	
**Highest level of education**		0.005		0.205		0.158
High School or less	Reference		Reference		Reference	
Some college	0.76 (0.39-1.47)		1.07 (0.61-1.85)		0.53 (0.25-1.09)	
Bachelor degree	0.51 (0.27-0.98)		1.48 (0.85-2.55)		0.54 (0.26-1.11)	
Any graduate school	0.43 (0.22-0.83)		1.30 (0.75-2.26)		0.44 (0.21-0.91)	
**U.S. Census region**		0.645		0.766		0.785
Midwest	0.99 (0.63-1.55)		0.92 (0.60-1.39)		0.90 (0.56-1.44)	
Northeast	0.93 (0.61-1.43)		0.80 (0.54-1.21)		0.79 (0.51-1.24)	
South	1.17 (0.79-1.73)		0.91 (0.63-1.31)		0.90 (0.60-1.37)	
West	Reference		Reference		Reference	
**Topical Modalities**						
	Penis gel, before		Penis gel, after		Rectal gel, before	
**Race/Ethnicity**		0.037		0.115		0.002
White, not Hispanic	Reference		Reference		Reference	
Black, not Hispanic	6.59 (1.54-28.24)		2.82 (1.05-7.59)		4.28 (1.73-10.61)	
Hispanic	1.27 (0.81-2.00)		1.32 (0.85-2.06)		1.45 (0.97-2.17)	
Other	1.65 (0.76-3.59)		0.83 (0.40-1.70)		1.85 (0.96-3.56)	
**Age (years)**		0.056		0.712		0.680
18-24	1.61 (1.06-2.45)		0.99 (0.65-1.50)		0.88 (0.59-1.31)	
25-29	1.16 (0.75-1.79)		0.86 (0.56-1.34)		0.83 (0.54-1.26)	
30-34	Reference		Reference		Reference	
**Highest level of education**		0.342		0.851		0.073
High School or less	Reference		Reference		Reference	
Some college	1.03 (0.47-2.24)		0.80 (0.38-1.67)		0.62 (0.31-1.24)	
Bachelor degree	0.91 (0.43-1.93)		0.89 (0.43-1.83)		0.50 (0.25-0.98)	

Any graduate school	0.71 (0.33-1.51)		0.78 (0.38-1.61)		0.44 (0.22-0.88)	
**U.S. Census region**		0.894		0.979		0.470
Midwest	0.82 (0.49-1.36)		1.06 (0.64-1.73)		0.78 (0.48-1.25)	
Northeast	0.92 (0.56-1.50)		0.95 (0.59-1.53)		0.73 (0.46-1.14)	
South	0.90 (0.57-1.41)		0.97 (0.63-1.50)		0.91 (0.60-1.38)	
West	Reference		Reference		Reference	
**Topical Modalities, continued**						
	Rectal gel, after		Suppository, before		Suppository, after	
**Race/Ethnicity**		0.137		0.057		0.548
White, not Hispanic	Reference		Reference		Reference	
Black, not Hispanic	2.52 (1.14-5.58)		2.69 (1.26-5.77)		1.28 (0.60-2.71)	
Hispanic	1.03 (0.69-1.54)		1.30 (0.86-1.97)		0.81 (0.54-1.22)	
Other	1.25 (0.66-2.35)		1.15 (0.61-2.17)		1.22 (0.68-2.22)	
**Age (years)**		0.254		0.691		0.113
18-24	0.85 (0.57-1.26)		0.87 (0.57-1.31)		0.73 (0.49-1.09)	
25-29	0.70 (0.46-1.08)		0.83 (0.53-1.29)		0.63 (0.41-0.97)	
30-34	Reference		Reference		Reference	
**Highest level of education**		0.041		0.266		0.735
High School or less	Reference		Reference		Reference	
Some college	0.81 (0.40-1.63)		0.62 (0.32-1.21)		0.76 (0.39-1.49)	
Bachelor degree	0.54 (0.27-1.07)		0.53 (0.27-1.02)		0.73 (0.38-1.41)	
Any graduate school	0.52 (0.26-1.05)		0.55 (0.28-1.07)		0.85 (0.44-1.65)	
**U.S. Census region**		0.845		0.784		0.446
Midwest	1.10 (0.68-1.77)		0.83 (0.51-1.37)		0.73 (0.46-1.18)	
Northeast	0.91 (0.58-1.42)		0.82 (0.51-1.31)		0.73 (0.47-1.14)	
South	1.05 (0.70-1.59)		0.96 (0.63-1.46)		0.75 (0.50-1.12)	
West	Reference		Reference		Reference	

[a]Compares respondents that are “somewhat likely” or “very likely” to respondents that indicated they are “somewhat unlikely” or “very unlikely” to use each modality. Responses that were “neither likely or unlikely” were set to missing. Wald chi-square type 3 *P* values were used with an alpha=0.05.

[b]Every 1-3 months

[c]Two pills 24 hours before sex and two one-pill doses after.
